# Levosimendan in trauma patients with acute cardiac failure

**DOI:** 10.1186/cc9514

**Published:** 2011-03-11

**Authors:** AN Afonin, N Karpun

**Affiliations:** 1 Burdenko Main Military Clinical Hospital, Moscow, Russia

## Introduction

Acute heart failure (AHF) is common among trauma patients with pre-existing coronary artery disease (CAD) and myocardial perfusion defect. The therapy is aimed at increased contractility while decreasing afterload and includes β_1_-adrenergic agents and phosphodiesterase III inhibitors, which act by increasing the intracellular calcium (Ca) concentration, thus markedly increasing myocardial energy consumption and risk of arrhythmias. The new Ca sensitizer levosimendan enhances cardiac performance without increasing myocardial energy demand and oxygen consumption. We report new use of levosimendan in polytrauma victims with AHF.

## Methods

In this prospective randomized clinical trial we studied effects of levosimendan on myocardium of polytrauma victims with a history of CAD who subsequently developed AHF as diagnosed by invasive monitoring and transthoracic echocardiography. Dobutamine was administered initially to maximum dose or effect and later combined with levosimendan (Group I, *n *= 12) or with adrenaline (Group II, *n *= 14). The hemodynamic data were recorded every 6 hours. The primary outcome measures were ECG, cardiac index (CI), troponin I (TnI), and incidence and type of complication. The secondary measures were global perfusion indices: atrial natriuretic peptide (ANP), serum lactate (SL), and inotropic therapy duration.

## Results

A second inotropic drug infusion was added when AHF persisted with average CI of 2.1 ± 0.15 l/minute/m^2 ^and left ventricular ejection fraction of 41 ± 7% despite achieved normovolemia (CVP 11 ± 2 mmHg, pulmonary artery wedge pressure 15 ± 1 mmHg) and continued dobutamine infusion to the maximum effective dose. CI improved to 3.5 ± 0.14 and 2.6 ± 0.33 l/minute/m^2 ^in Groups I and II, respectively (*P *< 0.03). Group I patients had lower levels of TnI, and rate of arrhythmias. ANP was significantly lower in Group I, as well as SL. Duration of inotropic therapy was 71 ± 10.5 hours in Group I and 102 ± 13.5 hours in Group II (*P *= 0.001).

## Conclusions

Levosimendan effectively enhances myocardial contractility and improves global circulation in polytrauma patients with refractory AHF. It had a significantly lower rate of complications related to increased work of the heart compared with what is usually reported with the use of catecholamines.
